# The New Departure Again—Letter from Prof. Henry S. Chase

**Published:** 1880-01

**Authors:** 


### EDITORIAL, ETC.

*The New Departure Again.*—We had occasion in the last JOURNAL to make brief comment upon that part of the report of Section VI, Metallurgy and the Chemistry of the Metals, (Maryland and District of Columbia Dental Association,) and adverted to the, as we thought, new theory—that of "destructive alkalinity"—broached by the authors of that report.

The following note, which we print with pleasure, sets those interested right upon the record, at least so far as gold is concerned; and we hasten to assure Dr. Chase that no intention to misrepresent the "New Departure" was intended.—EDS.

THE "NEW DEPARTURE" CORRECTS SOME ERRORS.

"1.—That pure gold introduced into a tooth as a filling is inert, and of itself exercises no electric or galvanic action on the tooth."

*Messrs Editors.*—On page 378 of December number of the AMERICAN JOURNAL OF DENTAL SCIENCE will be found the above quotation. You say on same page, "these statements and conclusions are in broad variance with the 'New Departure.'"

You are mistaken. The "New Departure" heartily agrees with the quotation. The apostles of the "New Departure" never denied the proposition, or said anything to the contrary.

It is very strange that the "New Departure" is continually misrepresented. We are getting rather tired of correcting the misrepresentations.

I will make a "New Departure" addition to the quotation, which will then read thus:

1.—That pure gold introduced into a tooth as a filling is inert and *of itself* exercises no electric or galvanic action on the tooth.

But if the gold-filled tooth is immersed in an acidulous fluid, there is, instantly, galvanic action set up, which must, according to the laws of electric action, disintegrate the tooth, as long as the above conditions continue.

We say that there is no destructive action on the tooth while it is bathed in neutral oral fluids.

Furthermore, we do not advocate the use of amalgams which leak. There are those which do not leak; these we use.

Furthermore, we do not advocate the use of plastic fillings in which Chlorine forms a part.

We know that the "Oxy-phosphates" are far better, and advocate their use.

Furthermore, we never have stated that plastic fillings are permanent.

We do believe in and advocate the use of plastics for the purpose of tooth salvation, even if the fillings need frequent renewal on their exposed surfaces.

We have found by numerous experiments that a cube of dentos with a large gold plug in it will dissolve six times as much in a given time as a similar cube not plugged at all, when both are placed in dilute vinegar, or lemon juice. When plugged with Amalgam, four times as much; Tin, three times as much; Gutta-percha, one time as much; Oxy-phosphates, no loss of dentos.

In the latter case the electric action is reversed. In this case the plug is positive to the dentos, and so, of course, the plug loses in weight while the dentos is preserved.

With metallic plugs the cubes of dentos were positive to the plugs, and of course had to lose substance. In a battery it is the positive element that loses weight.

Any dentist who will take the trouble to study electricity and galvanism, and then make experiments with dentos and filling materials, cannot come to any other conclusion than that *the Principles of the New Departure are true*, and are in harmony with electrical science.

HENRY S. CHASE,
St. Louis, Mo.

